# Epitope Mapping Immunoassay Analysis of the Interaction between β-Amyloid and Fibrinogen

**DOI:** 10.3390/ijms20030496

**Published:** 2019-01-24

**Authors:** Vo Van Giau, Seong Soo A. An

**Affiliations:** Department of Bionano Technology, Gachon University, Seongnam, 1342 Sungnamdaero, Sujeong-Gu, Seongnam, Gyeonggi 461-701, Korea; giauvvo@gmail.com or giauvvo@gachon.ac.kr

**Keywords:** Alzheimer’s disease, amyloid-β, epitope mapping, ligand, fibrinogen

## Abstract

The vast majority of patients with Alzheimer’s disease (AD) suffer from impaired cerebral circulation. Substantial evidence indicates that fibrinogen (Fbg) and fibrin clot formation play an important role in this circulatory dysfunction in AD. Fbg interacts with β-amyloid (1-42) (Aβ), forming plasmin-resistant abnormal blood clots, and increased fibrin deposition has been discovered in the brains of AD patients and mouse models. In this study, biochemical approaches and the epitope mapping immunoassay were employed to characterize binding epitopes within the Fbg and complementary epitopes in Aβ. We discovered the Aβ5–25 peptide as the most critical region for the interaction, which can be inhibited by specific monoclonal and polyclonal antibodies against the central region of Aβ. Aβ binding to Fbg may block plasmin-mediated fibrin cleavage at this site, resulting in the generation of increased levels of plasmin-resistant fibrin degradation fragments. Our study elucidates the Aβ–Fbg interaction that may involve the mechanism by which Aβ–Fbg binding delays fibrinolysis by plasmin, providing valuable information in the development of therapeutic approaches for AD.

## 1. Introduction

Alzheimer’s disease (AD) is a neurodegenerative disease in which vascular pathology plays a vital role [1,2,3,4]. The most important and well-known pathological features of AD are extracellular β-amyloid (1–42) (Aβ) plaques, intracellular tau tangles, neuroinflammation, and neuronal loss [5]. Fibrinogen (Fbg) is a large glycoprotein, composed of two fragment D domains and one fragment E domain, which consist of a heterodimer composed of pairs of α, β, and γ chains [6]. There are several functional consequences of binding of Aβ to Fbg, which induces a structural change in the C-terminal region of the Fbg β-chain (β384–393) [7] and results in the formation of fibrin with increased resistance to fibrinolysis [7,8]. Accumulating evidence implicates Fbg, the main protein component of blood clots, in the pathogenesis of AD. Hence, disturbances to fibrinolysis may have significant consequences for occlusive and inflammatory pathology in various diseases [3] including AD [9]. Indeed, many studies suggested that the interaction between Fbg and Aβ resulted in forming plasmin-resistant abnormal blood clots, which may be increased fibrin deposition in the brains of AD patients and mouse models [8,10,11,12]. Fibrin clots can contribute to the pathology of AD by forming occlude capillaries and restricting blood flow. Furthermore, previous studies demonstrated that the Aβ was a factor capable of modulating fibrin clot structure and stability [7,13]. Aβ42 bound Fbg with a Kd of 26.3 ± 6.7nM7, as well as fibrin clots formed in the presence of Aβ42, are structurally altered and more resistant to fibrinolysis. Aβ42 can also bind to pre-formed fibrin and block the access of plasmin to fibrin [7]. Over time, this could lead to disruption of microinfarcts and the blood–brain barrier, which are pathologies commonly observed in AD. To improve selectivity and potency of therapeutics against the Aβ–Fbg interaction, a better understanding of their interaction is needed.

In the present study, we discovered the regions within Aβ responsible for Aβ–Fbg binding using biochemical approaches and the epitope-mapping enzyme-linked immunosorbent assay (ELISA).

## 2. Results

### 2.1. The Preparation of Fbg and Aβ42 EpiMap ELISA Tests

The EpiMap ELISA was prepared from an array of synthetic peptides on 96-well microtiter plates using 14 overlapping peptides (15 mer) from the N- to C-terminus of the Aβ42 sequence (Figure 5). Overlapping peptides were synthesized with a cysteine residue at the N- or C-terminal ends to conjugate on the maleimide-activated microplates through their free sulfhydryl group. Increased hydrophobicity of overlapping peptides was encouraged with the addition of a cysteine. Unfortunately, the Aβ peptide no. 15 could not be purified due to its aggregation upon release from the solid-phase peptide synthesis beads. The overlapping peptides were conjugated to a maleimide-activated microplate through a cysteine residue. A schematic explanation of the overlapping peptide conjugation as well as the basic principle of the EpiMap ELISA test is described in Figure 6.

### 2.2. The Evaluation of Specificity and Sensitivity of the Antibodies

**Figure 1:**
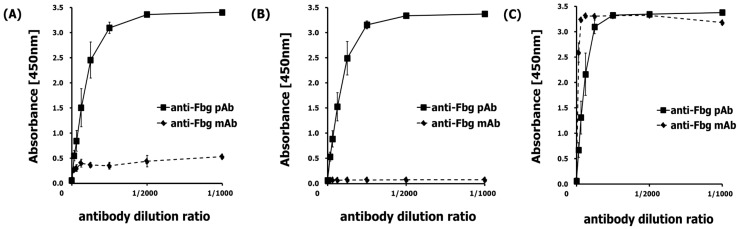
Each anti-Fbg, pAb and mAb, was tested using the indirect ELISA system. (**A**) At an Fbg-coated plate, the pAb had good affinity while the mAb had poor affinity. (**B**) At an Fbg fragment E-coated plate, the pAb had good affinity while the mAb had no affinity. (**C**) At an Fbg fragment D-coated plate, both the pAb and the mAb had good affinity.

The specificity and sensitivity of the antibodies used in this study, polyclonal rabbit anti-Fbg (pAb) and monoclonal anti-Fbg 85D4 (mAb), were checked with the indirect ELISA system. The pAb had an affinity to Fbg including its fragments D and E, while mAb, capturing only a specific conformational epitope in Fbg fragment D, only had an affinity for Fbg fragment D. Interestingly, Fbg itself showed little binding with mAb even though it contained the Fbg fragment D detected by mAb (Figure 1). Previous results show that the interaction between Aβ42 and Fbg or fragment D promotes Aβ42 fibrillization [13,14], which may account for the conversion of the oligomeric species of Aβ42 seen when incubated alone into fibrils in the presence of fragment D.

### 2.3. Protein Epitope Mapping with the EpiMap ELISA

**Figure 2:**
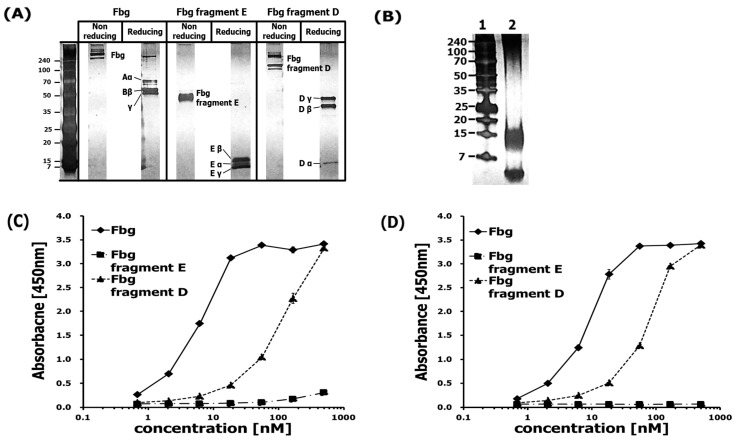
Aβ42 binds to Fbg and its fragments. (**A**) Tris-tricine gel electrophoresis result for Fbg and its fragments D and E. (**B**) Tris-tricine gel electrophoresis result for: (1) protein marker, and (2) Aβ42. (**C**) Binding of Fbg and its fragments was detected by the pAb. (**D**) Binding of Fbg and its fragments was detected by the mAb.

Our ELISA-based protein interaction assay was designed to investigate the interaction between Fbg and Aβ. Binding of Fbg at Aβ-coated plates was detected by both the pAb and the mAb. Fbg (340 kDa) is composed of 2 sets of 3 polypeptide chains (Aα: 67 kDa, Bβ: 52 kDa, γ: 46.5 kDa), its fragment E (~50 kDa) is composed of truncated small chains (Eα: 11 kDa, Eβ: 14 kDa, Eγ: 7 kDa), and its fragment D (85 kDa) is composed of truncated longer chains (Dα: 12 kDa, Dβ: 37.6 kDa, Dγ: 41 kDa). All three complexes were identified by a published gel electrophoretic method [15] (Figure 2A). At 500 μg/mL in 100 mM carbonate–bicarbonate buffer, the Aβ stock solution was composed of monomers (4 kDa), oligomers (8–16 kDa), and fibrils (approximately >70 kDa) (Figure 2B). Although a high-concentration solution of Aβ was used for electrophoresis due to the high detection limit of that assay, the Aβ used to coat ELISA plates had been diluted to 2 μg/mL in the carbonate–bicarbonate buffer, which would have lowered the degree of polymerization. The pAb interacted with Fbg and Fbg fragment D in proportion to their concentrations, while rarely interacting with Fbg fragment E even at high concentrations (Figure 2C). Similar to the pAb, the mAb interacted with both Fbg and Fbg fragment D in proportion to their concentrations, while no interaction between the mAb and Fbg fragment E could be detected (Figure 2D). These findings are similar to previous studies [13,14] that found Ab42 binding to fibrin(ogen) delays fibrinolysis by interfering with the binding of plasminogen and plasmin to fibrin [7] and that Ab42 binds to the β-chain of Fbg fragment D.

**Figure 3:**
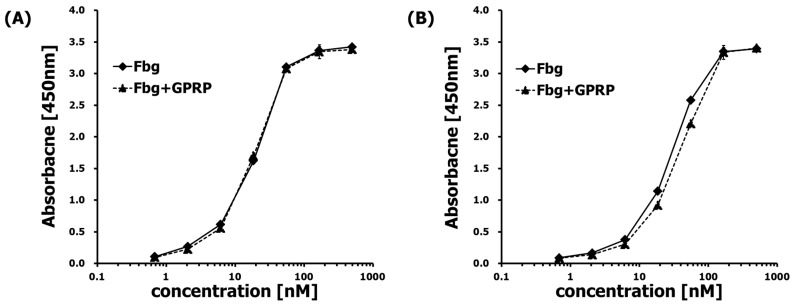
Aβ42 binds to Fbg and Fbg-GPRP peptide (1:50 molar ratio). (**A**) detecting by polyclonal rabbit anti-Fbg and (**B**) monoclonal anti-Fbg 85D4.

To further characterize the differential effect of the Fbg and Fbg-GPRP to the binding of the epitope versus the monoclonal and polyclonal antibodies, indirect ELISA was performed. Figure 3 shows that there was not a significant difference in the use of these two antibodies to detect the binding of Fbg and Fbg-GPRP peptide on Aβ42. This interaction study provided additional confirmation of the ELISA study, indicating that either pAb or mAb could have an affinity to the Fbg and Fbg-GPRP. 

### 2.4. Fbg Binding Analysis Using Aβ42 Epitope-Mapping ELISA

**Figure 4:**
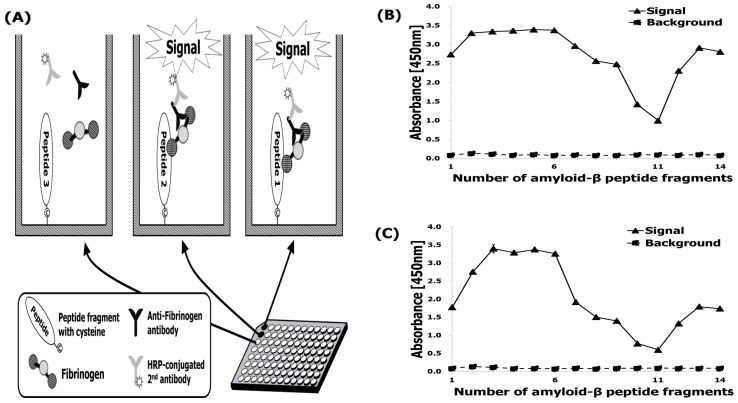
Aβ42 epitope-mapping ELISA to find a specific site interacting with Fbg. (**A**) Aβ42 epitope mapping to detect the site of interaction with Fbg. When Fbg bound to a specific sequence in Aβ42, the corresponding peptide- Fbg complex was detected by anti- Fbg antibody. (**B**) Fbg attached to the Aβ42 fragments detected by the pAb. (**C**) Fbg attached to the Aβ42 fragments detected by the mAb. Straight line: 100 μL of 20 μg/mL Fbg incubated with the antibody for 2 h at 25 °C before the Fbg detection step; dotted line: 100 μL of buffer incubated with the antibody for 2 h at 25 °C before the Fbg detection step. The straight line indicates non-specific binding of anti-Fbg antibodies.

To determine which region of Aβ42 is responsible for Fbg binding, fourteen Aβ fragments (numbered 1–14) were analyzed for their ability to bind Fbg and its fragments by monoclonal and polyclonal antibodies assay (Figure 4A). Among the 14 fragments, only Aβ5–25 showed binding to both pAb (Figure 4B) and mAb (Figure 4C) at N-terminus regions. 

**Figure 5:**
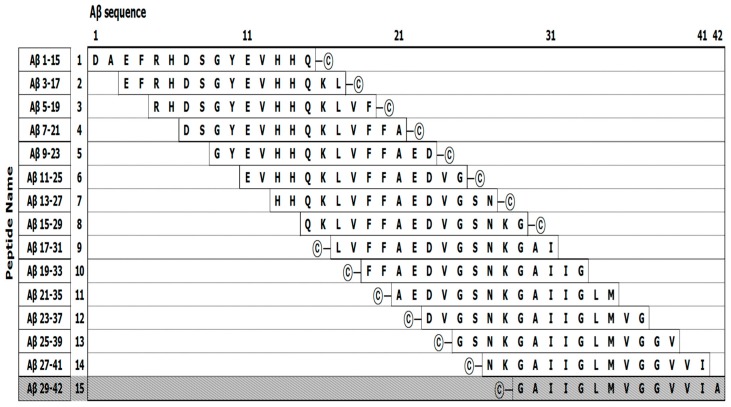
Schematic figures of Aβ_1–42_ (extracellular β-amyloid 1–42) overlapping peptides. Each fragment was assigned an ID number, and © indicates the cysteine residue used for peptide conjugation.

However, these figures were recorded with a relatively lower affinity to both fragments with numbering 10 and 11. Meanwhile, an unintended interaction between Aβ_42_ fragments and the anti-Fbg antibodies was not observed. Among the fragments #2 ~ #6, the consensus sequence corresponded to Aβ42 residues 5–25, as presenting Arg-His-Asp-Ser-Gly-Tyr-Glu-Val-His-His-Gln-Lys-Val-Phe-Ala-Glu-Asp-Val-Gly (Figure 5). In both the monoclonal and polyclonal antibodies, all 19 amino acid residues of the epitope were observed to be essential for the binding, consistent with the high selectivity of the antibodies. In contrast, the Aβ42 sequence 21–33, which is the consensus sequence for fragments #10 and #11, revealed relatively low affinity for Fbg (Figure 4C), which may be due to a loss of affinity of the fragments for those binding sites [14]. 

## 3. Discussion

Aβ associates with Fbg in vivo and in vitro, which alters the structure of the fibrin clot that is formed [8,10,11,12]. Since this interaction may have implications for AD pathogenesis, it is critical to gain a better understanding of the biochemical details. In addition, it will be important to which residues of Aβ are involved in this interaction by analyzing the binding of Fbg to regions of the Aβ peptide. We examined the Fbg-binding region within Aβ42 using an epitope mapping immunoassay analysis. Competitive inhibition of the Aβ–Fbg interaction showed that Aβ5–25 (Figure 4) had higher inhibitory efficacy than Aβ1–4 and Aβ26–42, suggesting that the peptide may play a physiological role in modulating Aβ42-mediated effects on fibrin clots. Previously, Aβ17–42 exhibits enhanced aggregation relative to full-length [14], which may be another beneficial consequence of enhancing a-secretase activity as a therapeutic strategy for AD Aβ42 [16]. Additionally, our evidence suggests that the Aβ–Fbg interaction might proceed by a new mechanism independent of the known Fbg knob-hole interaction. These previous findings supported a basis for the present study, which was focused on the identification of the essential amino acids within the Aβ4–10 [17] and Aβ17–40 [14] and Aβ17–42 [14] epitope sequences, and on the evaluation of their relative contribution to the interaction with Aβ-specific mono- and polyclonal antibodies [18]. 

One possible explanation for this is that although the N-terminus of Aβ42 may be directly involved in the interaction between Aβ42 and Fbg, the N-terminal residues of Aβ5–25 increase the stability of Aβ’s tertiary structure and promote its oligomerization, which may promote Aβ–Fbg binding to the Aβ fragment with strong affinity to Fbg. Overall, our results suggest that Fbg interacts with the central region of tertiary structured Aβ42, which is stabilized by its N-terminal residues. Our results also suggest that targeting the stable of Aβ42 via its fragment of N-terminal at 5–25 residues may be an alternative strategy for binding Aβ42–Fbg binding. 

Furthermore, we also demonstrated that Fbg fragment D contributed predominantly to the interaction, whereas fragment E showed little contribution. Using an mAb for Fbg detection, two experiments were conducted, one with and one without Aβ. In the absence of Aβ, a lower binding profile of the mAb to Fbg was measured because of the Fbg molecules being in their native conformation, but a higher binding tendency was detected in the presence of Aβ because Aβ binding changed the Fbg conformation, resulting in exposure of a preferred conformational epitope for mAb binding. Epitope mapping of Aβ demonstrated that Fbg possesses a higher binding affinity to the soluble N-terminus of Aβ. Consistent with this, the Aβ oligomer, which has the N-termini exposed and the C-termini buried in the center of the structure [19,20], seems to have a close relationship to the pathology of Aβ–Fbg interaction. Additionally, a fibrin clot formed in the presence of Aβ had unusual fibrinolysis resistance [8]. Aβ–Fbg interaction might be the cause of the chronic inflammation seen in AD. Fbg possesses a versatile binding capability [21] and many studies have focused on its interactions. For example, the α_IIb_β_3_ integrin of platelets [22] and mast cells [23] was able to bind to Fbg. 

Moreover, inflammation-related proteins such as toll-like receptor-4 [24], Mac-1 [25], and CD11c/CD18 [26] interact with Fbg. Fbg, having multiple binding sites, might also bind with Aβ fragments as well, forming a signaling pathway by which Aβ could, in principle, potentiate inflammatory processes. The interaction of residues 366–414 of the Fbg β chain with Aβ has been studied, and it is reported that this sequence is located in proximity to the β hole in Fbg fragment D [13]. In addition, it was also reported that at high molar ratio, GPRP peptide can bind to the Fbg β hole [27]. In the present study, the treatment of Fbg with GPRP peptide had little effect on its ability to bind Aβ (Figure 3), suggesting that the Aβ–Fbg interaction is independent of knob-hole interaction. One study suggested that the sequence of Fbg involved in binding to Aβ inhibits plasmin accessibility, and/or that Aβ attachment to Fbg leads to tight fibrin clot formation [7]. This binding model would be similar to the interaction between ApoE and Ab_42_ [28,29,30], which contributes to its selectivity and high avidity for Ab oligomers and fibrils, including aggregates composed of N-truncated Aβ variants, without targeting physiologic Ab monomers. A previous study demonstrated that the binding affinity of soluble Aβ oligomers to Fbg is nearly 10-fold higher than that of Aβ monomers [31]. These observations are possible clues as to the binding mechanism, but their interpretation is not yet clear. Fbg has previously been proposed as a potential biomarker for diagnosing AD [32]. Consistent with this, high concentrations of Fbg in the blood may lead to cognitive decline in mild-cognitive-impairment patients [33]. Moreover, a hyperhomocysteinemia showing cognitive decline resembling AD pathology was found to be caused by an Aβ–Fbg–collagen complex [34]. Some of the research on the relationship between Fbg and AD might lead to new therapeutic approaches [5,31,35]. 

## 4. Materials and Methods

### 4.1. Materials

The whole sequence of the human Aβ42 peptide and its fragments were synthesized for epitope mapping by Lugen Sci Co., Ltd., Seoul, Korea. A dual-color, broad-range, pre-stained marker (7–240 kDa) was also purchased from Lugen. Fbg from human plasma, monoclonal anti-Fbg 85D4 (mAb), phosphate buffered saline (PBS), PBS containing TWEEN^®^20 (PBST), 3,3′,5,5′ tetramethylbenzidine (TMB) liquid substrate system for ELISA, ethylenediaminetetraacetic acid (EDTA), l-cysteine, Tris base, and 2-mercaptoethanol were purchased from Sigma–Aldrich. Human Fbg fragment E, Pierce Protein-Free (PBF) blocking buffer, Pierce maleimide-activated plates, goat anti-rabbit IgG (H + L) secondary antibody, HRP conjugate, goat anti-Mouse IgG (H + L) secondary antibody, HRP conjugate, BupH carbonate–bicarbonate buffer, and Nunc-Immuno™ MaxiSorp™ Modules (flat bottom, 96-well format) were obtained from Thermo Scientific. Human Fbg fragment D, polyclonal rabbit anti-Fbg (pAb), and Probumin^®^ bovine serum albumin (BSA) were purchased from Millipore. Electrophoresis equipment and reagents were purchased from Bio-Rad Laboratories (Hercules, CA, USA). 

### 4.2. The Preparation of the Aβ42 Fragments and Aβ42 EpiMap ELISA Tests

Fifteen Aβ42 fragments for epitope mapping were synthesized as per our previous work [36]. Each fragment was composed of 15 amino acids (Figure 5). The fragments formed a series of sequences, each member of the series being shifted along the Aβ42 sequence by an interval of 2 amino acid residues from the previous member. Unfortunately, the peptides numbered 15 failed to be synthesized due to an aggregation problem. These peptides were generated with a cysteine at the end of the N-terminus of the numbered 1–8 and the C-terminus for the peptides 9–14 for linkage to maleimide-activated plates. The interval of 2 amino acids between fragments resulted in a high resolution of the epitope mapping result. 

**Figure 6:**
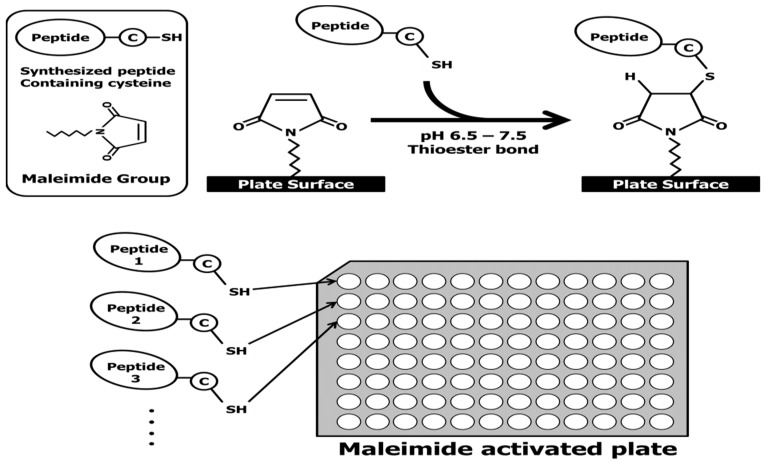
Synthetic Aβ42 fragments were coated on maleimide-activated plates and the principle of epitope-mapping enzyme-linked immunosorbent assay (ELISA) applied.

Each high-concentration synthetic Aβ fragment solution in dimethyl sulfoxide (DMSO) was first diluted in a PBS buffer (pH 4.5) at a 1:1 volume ratio. These solutions were then further diluted to 25 μg/mL in the PBS buffer (pH 7.2) containing 10 mM EDTA. Each resulting solution was dispensed into Pierce maleimide-activated plates at 100 μL per well and incubated overnight at 4 °C (Figure 6). After 3 PBST washes, unreacted maleimide groups were blocked by adding 200 μL of 25 μg/mL L-cysteine solution in PBS and incubating at 25 °C for 1 h. After 3 PBST washes, 100 μL of 20 μg/mL Fbg in PBS + 1% BSA were aliquotted into the wells and incubated at 25°C for 2 h. After 3 PBST washes, 100 μL of detection antibody solution (pAb or mAb diluted 1:5000 in PBS + 1% BSA) were added to the wells and incubated at 25 °C for 1 h. After 3 PBST washes, HRP-conjugated secondary antibody was diluted 1:5000 in PBS and was added at 100 μL per well and incubated at 25 °C for 1 h. After 3 PBST washes, 100 μL of TMB substrate was added and incubated at 37 °C for 25 min. The TMB reaction was stopped by adding 50 μL of 2NH_2_SO_4_. Absorbance at 450 nm was measured with a Victor 3 Perkin–Elmer plate reader. Experimental data were reported as the mean ± S.E.M. of three replicates.

### 4.3. Fbg and Its Fragments Binding Assay Using the Anti-Fbg Antibody

**Figure 7:**
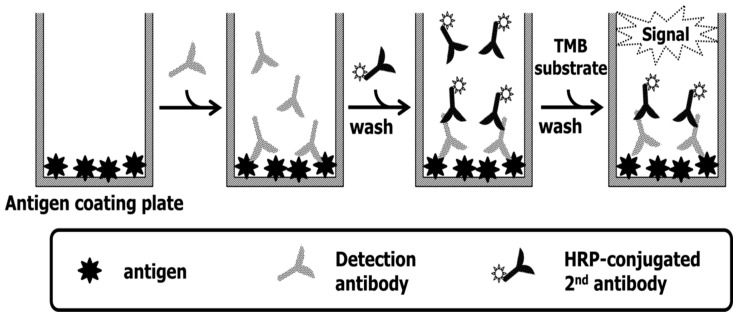
Schematic of indirect ELISA. The antigens were Fbg, Fbg Frag E, and Fbg Frag D. The detection antibodies were pAb and mAb.

An indirect ELISA system was used for the anti-Fbg antibody test as described Figure 7. Each of Fbg, Fbg fragment E, and Fbg fragment D was diluted to 2 μg/mL in 100 mM carbonate–bicarbonate buffer, dispensed into a Nunc transparent 96-well plate at 100 μL per well, and incubated at 4 °C overnight. After 3 PBST washes, the remaining reactivity of the wells was blocked using Pierce Protein-free blocking buffer at 25 °C for 1 h. After 3 more PBST washes, the plates were dried in a vacuum chamber for 2 h. Antibodies pAb from rabbit and mAb from mouse were serially diluted 1:1000, 1:2000, 1:4000, 1:8000, 1:16,000, 1:32,000, and 1:64,000 in PBS + 1% BSA, and 100 μL of each dilution were aliquotted into the wells. The plates were incubated for 1 h at 25 °C and washed 3 times with PBST. Each HRP-conjugated secondary antibody was diluted 1:5000 in PBS starting with a volume of 100 μL, then 100 μL of secondary solution was added to each well and incubated for 1 h at 25 °C. After 3 PBST washes, 100 μL of TMB substrate was added to each well and incubated at 37 °C for 25 min. The TMB reaction was stopped by adding 50 μL of 2NH_2_SO_4_ to each well. Absorbance at 450 nm was measured with a Victor 3 Perkin–Elmer plate reader. Experimental data were reported as the mean ± S.E.M. of three replicates. 

### 4.4. ELISA Aβ42–Fbg Interaction Assay

**Figure 8:**
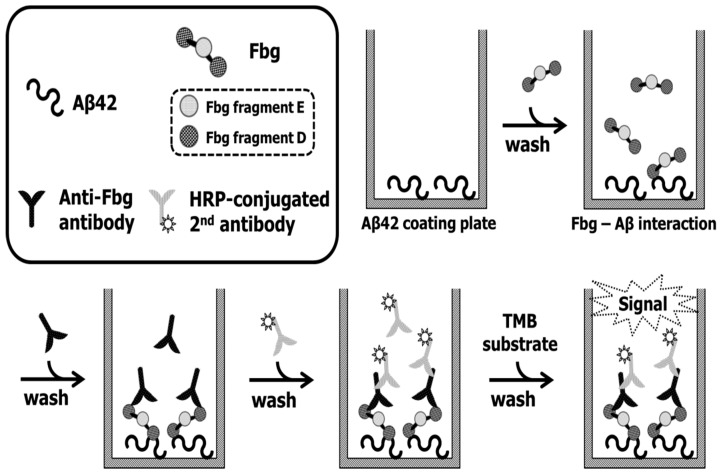
Scheme of the ELISA Aβ42–Fbg (fibrinogen) interaction assay.

A dilution of 2 μg/mL in 100 mM carbonate–bicarbonate buffer for 10 mg/mL of Aβ stock solution in DMSO and 100 μL/well were dispensed into a Nunc transparent 96-well plate and incubated at 4°C overnight. After 3 PBST washes, the remaining reactivity of the wells was blocked using Pierce protein-free blocking buffer at 25 °C for 1 h. After 3 PBST washes, the plate was dried in a vacuum chamber for 2 h. Fbg, Fbg fragment E, and Fbg fragment D were each serially diluted 1:1 in PBS + 1% BSA, starting with 0.5 μM and ending with 7.8125 nM. These protein solutions were dispensed into Aβ-coated plates at 100 μL per well and incubated at 25 °C for 2 h. After 3 PBST washes, detection antibody solutions (pAb or mAb diluted 1:5000 in PBS + 1% BSA) were added to the plates at 100 μL per well and incubated at 25 °C for 1 h. After 3 PBST washes, each well received 100 μL of HRP-conjugated secondary antibody diluted 1:5000 in PBS followed by incubation at 25 °C for 1 h. After 3 PBST washes, 100 μL of TMB substrate were aliquotted into each well and incubated at 37 °C for 25 min. The TMB reaction was stopped by adding 50 μL of 2NH_2_SO_4_ to the well. Absorbance at 450 nm was measured with a Victor 3 Perkin–Elmer plate reader as described in Figure 8. Experimental data were reported as the mean ± S.E.M. of three replicates.

To explore Fbg binding under conditions of inhibited Fbg polymerization, a 10 mg/mL stock solution of Gly-Pro-Arg-Pro (GPRP) peptide in DMSO was mixed with a 1 mg/mL stock solution of Fbg to produce a 50:1 GPRP:Fbg molar ratio. This solution was serially diluted in PBS + 1% BSA to 1/3 of the previous concentration at each step, starting with 0.5 μM and ending with 685 pM. These protein solutions were dispensed into Aβ-coated plates at 100 μL per well and incubated at 25 °C for 2 h. Further processing was the same as above.

### 4.5. SDS-Stable Complex Formation

Laemmli 4× sample buffers with and without 2-mercaptoethanol were prepared according to the manufacturer’s instructions (Sigma–Aldrich, St. Louis, MO, USA). Each 500 ng sample of Fbg, Fbg fragment E, or Fbg fragment D was mixed with each sample buffer at a 3:1 volume ratio, heated in boiling water for 3 min, and cooled in an ice bath. A 12% Mini-PROTEAN^®^ TGX™ gel was used for separation and a 1× Tris-glycine-SDS running buffer was filled in the gel top and bottom chambers. The samples were loaded, separated slowly, and silver stained.

Aβ42 was prepared as a 500 μg/mL solution in 100 mM carbonate–bicarbonate buffer. After a 4°C overnight incubation, a Aβ42 sample was mixed with Tris-tricine sample buffer at a 1:2 volume ratio, heated for 3 min in boiling water, and cooled in an ice bath. A 10–20% Mini-PROTEAN^®^ Tris-tricine gel was used for separation. A 1× Tris-tricine-SDS cathode buffer was filled in the gel top chamber, and a 0.2 M Tris pH 8.9 buffer was filled in the bottom chamber. The 5 μg samples were loaded, separated slowly, and silver stained.

## 5. Conclusions

In conclusion, in the present study, we identified the amino acid residues (Aβ5–25) that are critical to the Aβ–Fbg interaction through specific epitope by monoclonal and polyclonal antibodies. The findings of this study also reveal that antibody epitopes within the center of Aβ are potentially important for plaque clearance and neuronal protection via an Fbg-mediated mechanism. These results may contribute the development of effective therapeutics against the Aβ–Fbg interaction to treat cerebrovascular abnormalities in AD.
